# Datasets comprising the quality validations of simulated protein-ligand complexes and SYBYL docking scores of bioactive natural compounds as inhibitors of *Mycobacterium tuberculosis* protein-targets

**DOI:** 10.1016/j.dib.2022.108146

**Published:** 2022-04-10

**Authors:** Sravan Kumar Miryala, Soumya Basu, Aniket Naha, Reetika Debroy, Sudha Ramaiah, Anand Anbarasu, Saravanan Natarajan

**Affiliations:** a Medical and Biological Computing Laboratory, School of Biosciences and Technology, Vellore Institute of Technology (VIT), Vellore 632014, Tamil Nadu, India; b Department of Biochemistry, ICMR-National Institute for Research in Tuberculosis (NIRT), Chennai 600031, India

**Keywords:** Docking, Simulation, Natural compounds, Tuberculosis, Therapeutics

## Abstract

Docking scores and simulation parameters to study the potency of natural compounds against protein targets in *Mycobacterium tuberculosis* (M*tb*) were retrieved through molecular docking and *in-silico* structural investigation. The molecular docking datasets comprised 15 natural compounds, seven conventional anti-tuberculosis (anti-TB) drugs and their seven corresponding M*tb* target proteins. M*tb* protein targets were actively involved in translation mechanism, nucleic acid metabolism and membrane integrity. Standard structural screening and stereochemical optimizations were adopted to generate the 3D protein structures and their corresponding ligands prior to molecular docking. Force-field integration and energy minimization were further employed to obtain the proteins in their ideal geometry. Surflex-dock algorithm using Hammerhead scoring functions were used to finally produce the docking scores between each protein and the corresponding ligand(s). The best-docked complexes selected for simulation studies were subjected to topology adjustments, charge neutralizations, solvation and equilibrations (temperature, volume and pressure). The protein-ligand complexes and molecular dynamics parameter files have been provided. The trajectories of the simulated parameters such as density, pressure and temperature were generated with integrated tools of the simulation suite. The datasets can be useful to computational and molecular medicine researchers to find therapeutic leads relevant to the chemical behaviours of a specific class of compounds against biological systems. Structural parameters and energy functions provided a set of standard values that can be utilised to design simulation experiments regarding similar macromolecular interactions.

## Specifications Table

**Table:** 

Subject	***Subject area:*** Biological Sciences ***Sub-section:*** Structural biology
Specific subject area	*In silico* structural analyses of protein-ligand complexes with molecular dynamics based on chemical interactions
Type of data	Tables and Figures
How the data were acquired	We selected seven conventional drug targets in *Mycobacterium tuberculosis* whose 3D structures were downloaded from the Protein Data Bank (PDB) database (https://www.rcsb.org/). The structural and functional domains of the proteins were screened from UniProt (https://www.uniprot.org/), InterPro (https://www.ebi.ac.uk/interpro/) and Pfam (http://pfam.xfam.org/) databases. The ligand structures were acquired from the National centre for Biotechnology Information (NCBI) PubChem compound database (https://pubchem.ncbi.nlm.nih.gov/) and Drug bank database (https://www.drugbank.com/). The absence of a ligand structure in drug repositories was compensated by drawing the same with the ChemSketch tool followed by 3D structures generation using OpenBabel online server (http://www.cheminfo.org/Chemistry/Cheminformatics/FormatConverter/index.html). The bond integrities were validated using the Avogadro tool. The datasets comprising molecular docking scores of natural compounds (ligands) and the classical anti-Tuberculosis drugs with their respective targets were generated using the SYBYL-Surflex-docking tool kit. The best-docked complexes were subjected to topology adjustments for individual proteins and ligands using CHARMM36-Mar2019 force-field-TIP3P water-model and CGenFF (https://cgenff.umaryland.edu/) online server with default parameters respectively. The integrated simulation suite GROMACS 2018.1 was utilized. The optimized macromolecular complexes were solvated by centering in an aqueous dodecahedron box of uniform edge distance of 1.0 nm. Subsequently, requisite counter ions (Na^+^ or Cl^−^) were added to balance the charges of the solvated system. Energy was minimized using integrated steepest descent algorithm for 50,000 steps and convergence-tolerance of 1000 kJ/mol nm^−1^. System equilibration with standard NVT (constant Number of particles, Volume and Temperature) and NPT (constant Number of particles, Pressure and Temperature) ensembles were performed for
	100 ps. A constant pressure of 0 (zero) bar and temperature of 300 K with uniform density of ∼1040 kg/m^3^ was set for parameterization. Final molecular dynamics simulation (MDS) was carried out for 75 ns. Grace software was employed to visualize the trajectories of simulation parameters. A chronological list of commands and other associated parameter files to run simulation along with the entire MD-simulation files have been provided in the associated Mendeley dataset folder as mentioned in subsequent sections.
Data format	Data is in raw and analysed form.
Description of data collection	The structural chemistry data was acquired from authorised databases and repositories, followed by necessary optimisations using licensed (academic and professional) software. The reported docking scores and simulation parameters are based on universally accepted terms/standards.
Data source location	• **Institution**: Vellore Institute of Technology, Vellore • **City/Town/Region**: Vellore, Tamil Nadu • **Country**: India
Data accessibility	Data is available within this article and the raw data files in excel format and other standard formats for simulation has been uploaded on public repository and datasets with active link below is provided as the supplementary data. ***Link***: (https://data.mendeley.com/datasets/94rh86jfpk/3), DOI: 10.17632/94rh86jfpk.3
Related research article	The presented dataset is associated with our recent publication mentioned below[1]: S. K. Miryala, S. Basu, A. Naha, R. Debroy, S. Ramaiah, A. Anbarasu & S. Natarajan (2021). Identification of bioactive natural compounds as efficient inhibitors against Mycobacterium tuberculosis protein-targets: A molecular docking and molecular dynamics simulation study. *Journal of Molecular Liquids*, *341*, 117,340. https://doi.org/10.1016/j.molliq.2021.117340

## Value of the Data

1) There are four distinct types of datasets presented in this manuscript:
  a) ***Raw docking scores*** like Crash score, G-score, PMF score, d-score, Chem scoresand C-scores can help understand different chemical factors affecting ligand-protein binding.
  b) ***Optimized protein-ligand complexes used for simulation*** will provide comprehensive idea about the fundamental format of input biomolecular structural complexes to run molecular dynamics simulations.
  c) ***The optimized molecular dynamics parameter files, output files and list of commands*** will definitely facilitate in further analysis and performing essential dynamics studies besides guiding researchers to replicate similar experimental approaches and objectives.
  d) ***Trajectories of optimised conditions for simulation*** regarding individual protein-ligand complexes can give a fair idea of the set of conditions required to simulate a specific type of biomolecule (protein) interacting with a certain class of compounds.
2) The datasets can be of interest to bioinformaticians, computational biologists, phytochemists and molecular medicine researchers, who can figure out leads relevant to the chemical behaviours of a certain class of compounds against biological systems.
3) The docking scores can further be exploited either based on individual compounds or collective understanding of a specific class of compounds or analysis of specific chemical parameters based on individual scoring algorithms.
4) The compounds that were not considered as per criteria presented in the main publication can further be explored similarly against other potent targets [1].
5) The optimised simulation parameters can readily guide researchers by providing a set of standard values that can be utilised to design simulation experiments regarding the same/similar macromolecules
6) The simulation profiles may encourage designing of efficient therapeutic agents by providing crucial interaction dynamics values.

## 1 Data Description

**Fig. 1:**
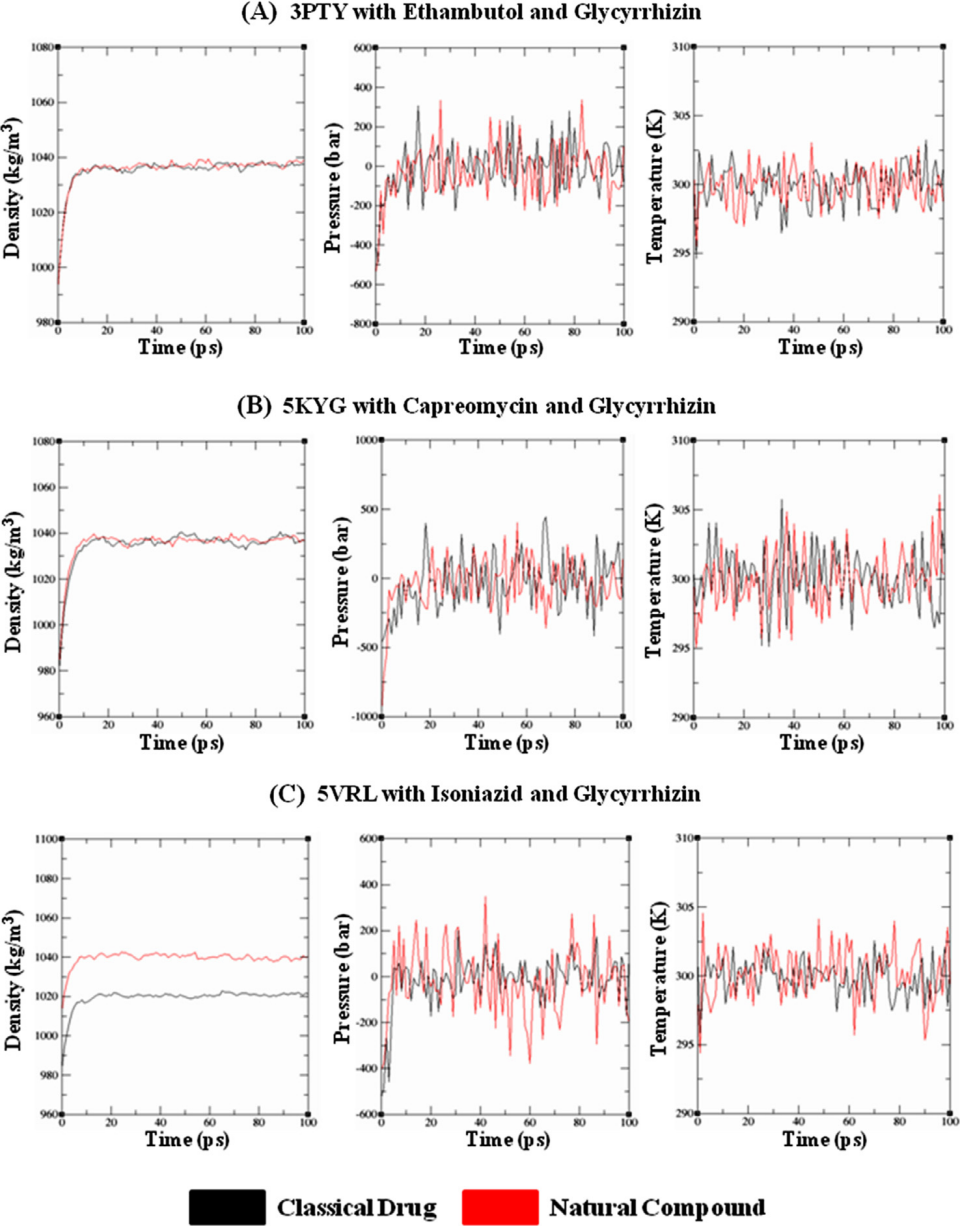
Quality check parameters after equilibrating the respective protein-ligand complexes prior to MD run depicting the Density gradients, Pressure and Temperature levels. **(A)** 3PTY with Ethambutol and Glycyrrhizin, **(B)** 5KYG with Capreomycin and Glycyrrhizin, **(C)** 5VRL with Isoniazid and Glycyrrhizin.

**Fig. 2:**
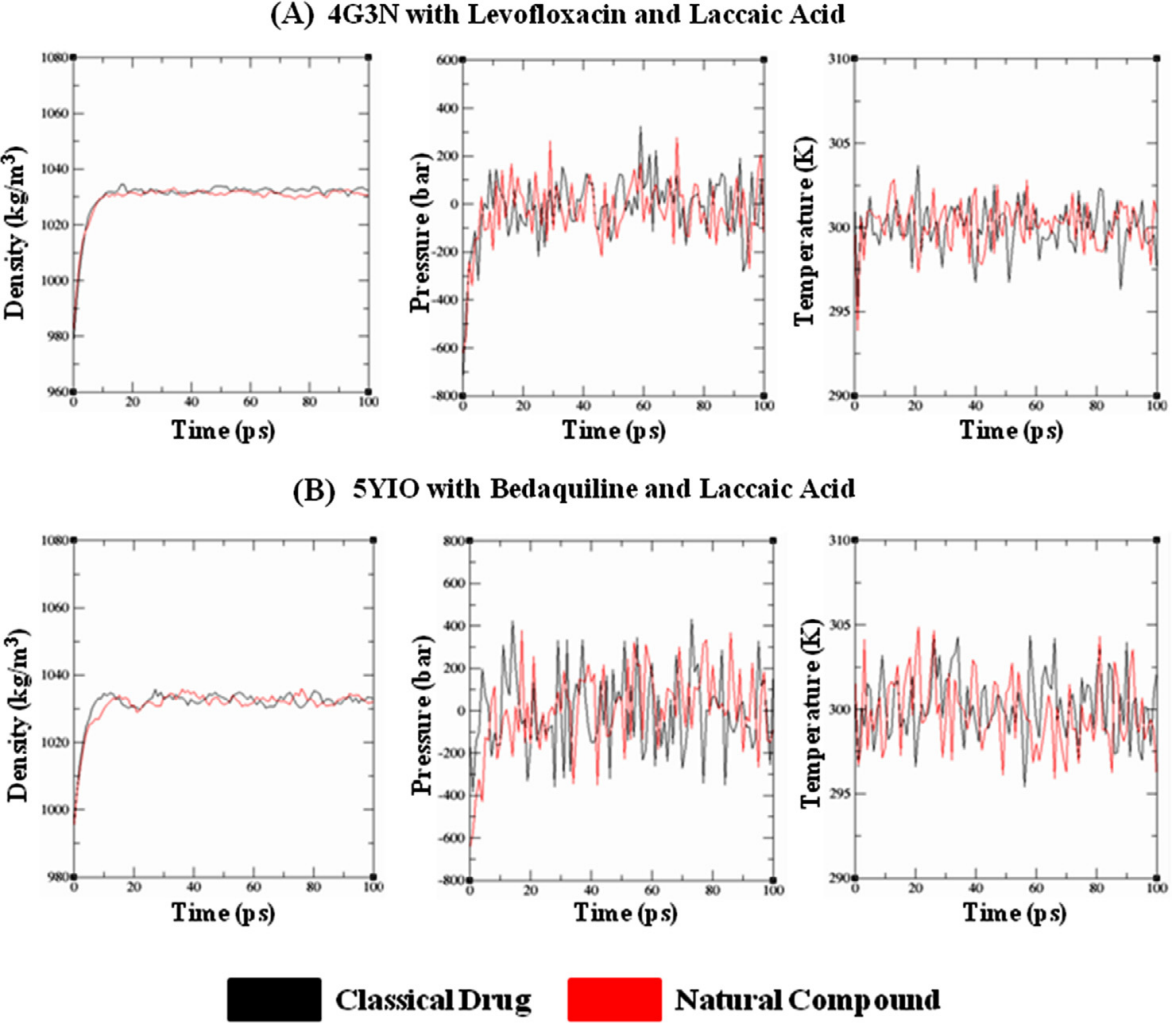
Quality check parameters after equilibrating the respective protein-ligand complexes prior to MD run depicting the Density gradients, Pressure and Temperature levels. **(A)** 4G3N with Levofloxacin and Laccaic Acid, **(B)** 5YIO with Bedaquiline and Laccaic Acid.

**Fig. 3:**
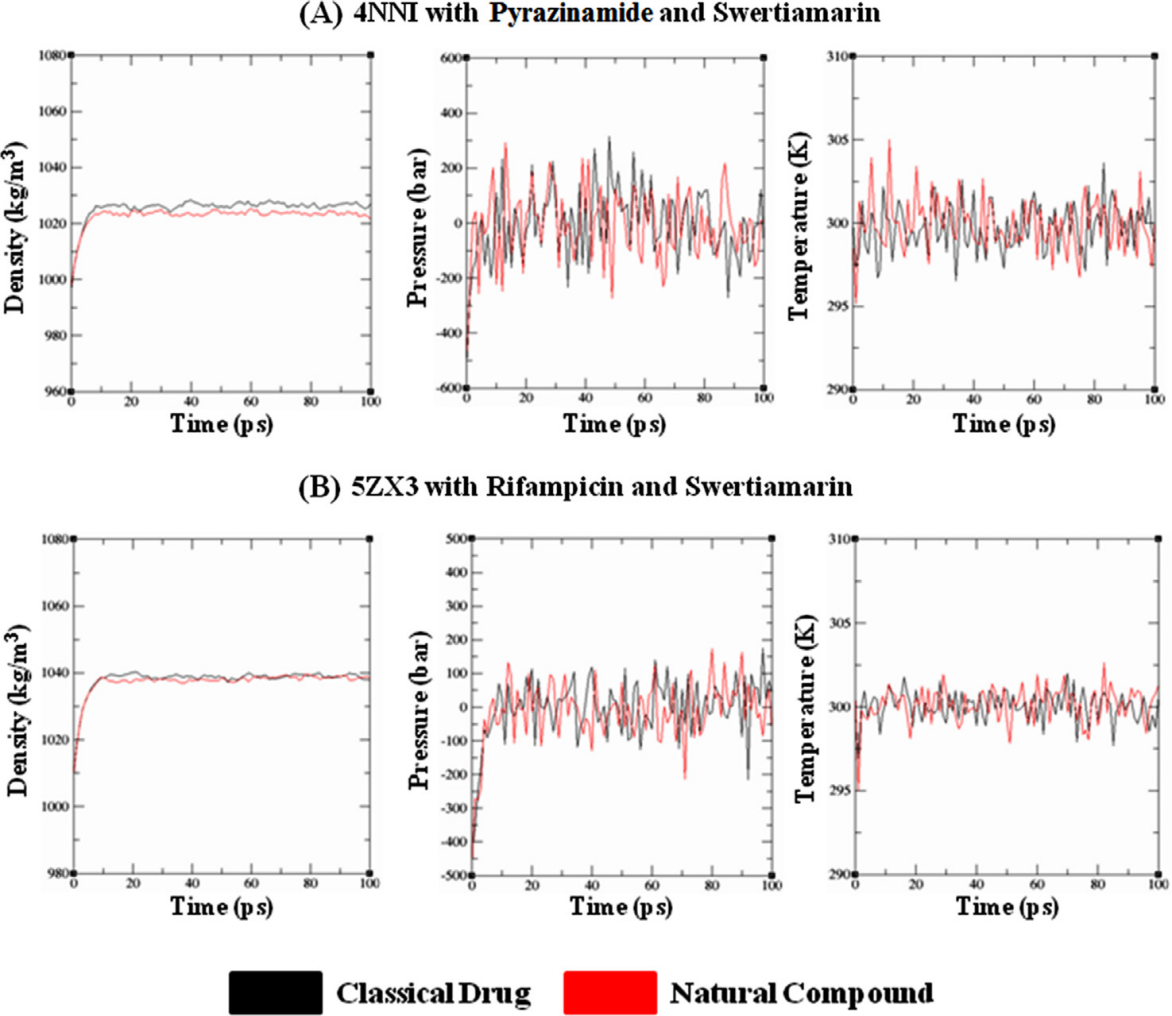
Quality check parameters after equilibrating the respective protein-ligand complexes prior to MD run depicting the Density gradients, Pressure and Temperature levels. **(A)** 4NNI with Pyrazinamide and Swertiamarin, **(B)** 5ZX3 with Rifampicin and Swertiamarin.

The presented datasets depict the feasibility of certain classes of natural compounds as therapeutic candidates against M*tb* protein targets. **Supplementary Files-1–7 (Docking_scores)** (https://data.mendeley.com/datasets/94rh86jfpk/3) portrayed the docking scores comprising crash score, G-score, PMF score, d-score, chem scores, polar, total score, consensus (C) score, number of Hydrogen-bonds of the natural compounds against M*tb* targets [Arabinosyl transferase (PDB ID: 3PTY); DNA Gyrase subunit A (PDB ID: 4G3N); Ribosomal protein S1 (PDB ID: 4NNI); 2′-O-Methyltransferase (PDB ID: 5KYG); Enoyl (acyl-carrier protein) reductase (PDB ID: 5VRL); F-ATP synthase epsilon chain (PDB ID: 5YIO) and RNA polymerase subunit C (PDB ID: 5ZX3)] as compared to respective classical drugs (Ethambutol, Levofloxacin, Pyrazinamide, Capreomycin, Isoniazid, Bedaquiline and Rifampicin). The optimized protein-ligand complexes used as input files for parameretization and simulation has been provided as **“MDS_input_files”** (https://data.mendeley.com/datasets/94rh86jfpk/3). The molecular dynamics parameter files along with the set of commands to run MDS are available as **“MDS_parameter_files”** (https://data.mendeley.com/datasets/94rh86jfpk/3). Fig. 1**-**3 represented the quality-check parameters after equilibrating certain protein-ligand complexes comprising classical and natural compounds prior to MD run depicting the electron Density, Pressure and Temperature levels [1]. The differences in molecular weight and number of atoms were reflected upon the electron density function of the individual protein-ligand complexes. The datasets for generating the figures has been provided explicitly in the **Supplementary Files-8–14 (Figure_datasets)** (https://data.mendeley.com/datasets/94rh86jfpk/3). The entire simulation dataset has been segmented appropriately based on the PDB IDs of the studied protein-targets. The different input and output files generated are made available under ``**MD_simulation_files**'' (https://data.mendeley.com/datasets/94rh86jfpk/3).

## 2 Experimental Design, Materials and Methods

Seven M*tb* proteins were selected, which are already targets of conventional anti-TB drugs [2]. Their 3D structures were obtained from RCSB-PDB (https://www.rcsb.org/), while the functional domains/motifs were obtained from InterPro (https://www.ebi.ac.uk/interpro/), Pfam (http://pfam.xfam.org/), and UniProt (https://www.uniprot.org/) databases. The classical drugs and the natural compounds were retrieved from the DrugBank (https://www.drugbank.com/) and PubChem Compound (https://pubchem.ncbi.nlm.nih.gov/) databases. ChemSketch tool [3] was employed in the absence of ligand structures for 2D structure construction followed by generation of 3D coordinates using the OpenBabel Chemical File Format Converter (http://www.cheminfo.org/Chemistry/Cheminformatics/FormatConverter/index.html). Further, the ligands were optimised with the Avogadro tool [4]. Molecular docking between the conventional anti-TB drugs and natural compounds with their respective targets was performed usingthe SYBYL-Surflex-docking tool kit (Tripos International, USA). The protein structures were refined to remove bound ligands and water molecules, fixing side chains, adding hydrogen atoms, followed by atomic-level charge designation using AMBER7 F99 force field. Thereafter, the proteins were energy minimised by Powell's method with Tripos force field followed by Protomol generation. Hammerhead functional scorings determined the polar, crash, entropic, hydrophobic and repulsive properties to yield the docked score datasets [5,6]. The MDS analyses for 75 nanoseconds (ns) was performed for each of the best-docked complexes with GROMACS 2018.1 suite [7]. Protein topologies were generated using CHARMM36-Mar2019 force-field mechanics and TIP3P model (for water cluster), while ligand topologies were built using CGenFF (https://cgenff.umaryland.edu/) online server with default parameters. The protein structures were placed within the center of the dodecahedron box of uniform edge distance of 1.0 nm, followed by solvation and addition of requisite counter ions (Na^+^ or Cl^−^) to the system. Steepest descent algorithm for 50,000 steps and convergence-tolerance of 1000 kJ/mol nm^−1^ were utilised for energy minimisation following system equilibration under standard NVT (constant Number of particles, Volume and Temperature) and NPT (constant Number of particles, Pressure and Temperature) ensembles for 100 ps [7], [8], [9], [10], [11], [12], [13], [14]. The trajectories of simulation parameters were visualised using Grace software (https://plasma-gate.weizmann.ac.il/Grace/).

## Ethics Statements

The work did not involve any human subjects, animal experiments and data from social media platforms.

## CRediT authorship contribution statement

**Sravan Kumar Miryala:** Data curation, Formal analysis, Visualization, Writing – original draft. **Soumya Basu:** Formal analysis, Visualization, Writing – original draft. **Aniket Naha:** Formal analysis, Visualization, Writing – original draft. **Reetika Debroy:** Formal analysis, Visualization, Writing – original draft. **Sudha Ramaiah:** Conceptualization, Methodology, Validation, Writing – review & editing. **Anand Anbarasu:** Funding acquisition, Conceptualization, Project administration, Supervision. **Saravanan Natarajan:** Funding acquisition, Conceptualization, Project administration, Supervision.

## Declaration of Competing Interest

The authors declare that they have no known competing financial interests or personal relationships that could have appeared to influence the work reported in this paper.

## Data Availability

Supplementary data related to the quality validations of simulated protein-ligand complexes and SYBYL docking scores of bioactive natural compounds as inhibitors of Mtb protein targets (Original data) (Mendeley Data).
